# Supplementary material for: Clinical characteristics and outcomes of SARS-Cov-2 B.1.1.529 infections in hospitalized patients and multi-surge comparison in Louisiana
Source: PLoS One. 2022 Oct 21;17(10):e0268853. doi: 10.1371/journal.pone.0268853 (PMC9586372; doi:10.1371/journal.pone.0268853)
Supplement: Attachment — Submitted filename: PONE-D-22-13503.pdf [file pone.0268853.s001.pdf]

PLOS ONE

Multi-surge comparison of COVID-19 characteristics and outcomes of hospitalized patients in Louisiana

--Manuscript Draft--

**Manuscript Number:**	PONE-D-22-13503
**Article Type:**	Research Article
**Full Title:**	Multi-surge comparison of COVID-19 characteristics and outcomes of hospitalized patients in Louisiana
**Short Title:**	Covid-19 surge comparison and outcomes
**Corresponding Author:**	Evan Rivere, MD<br>LSUHSC: Louisiana State University Health Sciences Center<br>Baton Rouge, LA UNITED STATES
**Keywords:**	COVID-19; SARS-CoV-2; Delta; Omicron; Public Health
**Abstract:**	Background<br>Peer reviewed data describing SARS-CoV-2 Omicron variant symptoms and clinical outcomes as compared to prior surges in the United States is thus far limited. We sought to determine disease severity, presenting features, and epidemiologic factors of the SARS-CoV-2 Omicron variant compared to prior surges.<br>Methods<br>Retrospective cohort analysis was performed on patients admitted during all five surges in Louisiana between March 2020 and January 2022. Patient data was pulled from the medical record, including a subset of patients during Surge 5 for individual chart review. Patients who were admitted to an FMOLHS hospital with a positive SARS-CoV-2 test during the 5 defined surge periods were included. Surges were compared using chi-squared tests and one way ANOVA for age, sex, vaccination status, length of stay, ICU status, ventilation requirement, and disposition at discharge. The records of patients admitted during the omicron surge were analyzed for presenting symptoms and incidental SARS-CoV-2 diagnosis.<br>Results<br>With each subsequent surge, a smaller proportion of patients presenting to the ED were admitted. Patients admitted during surge 5 had shorter lengths of stay and fewer comorbidities than prior surges. Fewer patients in surge 5 presented with a respiratory condition and fewer required ICU admission. In surges 4 and 5, fewer vaccinated patients were admitted compared to their unvaccinated counterparts. Overall mortality was lower in S5 (9%) than in S4 (15%). Of the SARS-Cov-2 positive patients in S5, 22.3% were felt to be incidental diagnoses.<br>Conclusions<br>As the COVID-19 pandemic progressed, a younger and less vaccinated population was at higher risk for severe disease, fewer patients required ICU admission and overall mortality decreased. Vaccinations have been protective for overall risk of hospitalization but once admitted did not confer additional protection against severe illness during the omicron surge. Age also played an important role in patient outcomes.
**Order of Authors:**	Evan Rivere, MD
**Additional Information:**	
**Question**	**Response**
**Financial Disclosure**<br><br>Enter a financial disclosure statement that describes the sources of funding for the work included in this submission. Review the submission guidelines for detailed requirements. View published research articles from *PLOS ONE* for specific	The author(s) received no specific funding for this work

Powered by Editorial Manager® and ProduXion Manager® from Aries Systems Corporation

examples.<br><br>This statement is required for submission and **will appear in the published article** if the submission is accepted. Please make sure it is accurate.<br><br>**Unfunded studies**<br>Enter: *The author(s) received no specific funding for this work.*<br><br>**Funded studies**<br>Enter a statement with the following details:<br>• Initials of the authors who received each award<br>• Grant numbers awarded to each author<br>• The full name of each funder<br>• URL of each funder website<br>• Did the sponsors or funders play any role in the study design, data collection and analysis, decision to publish, or preparation of the manuscript?<br>• **NO** - Include this sentence at the end of your statement: *The funders had no role in study design, data collection and analysis, decision to publish, or preparation of the manuscript.*<br>• **YES** - Specify the role(s) played.<br><br>* typeset	
**Competing Interests**<br><br>Use the instructions below to enter a competing interest statement for this submission. On behalf of all authors, disclose any competing interests that could be perceived to bias this work—acknowledging all financial support and any other relevant financial or non-financial competing interests.<br><br>This statement is required for submission and **will appear in the published article** if the submission is accepted. Please make sure it is accurate and that any funding sources listed in your Funding Information later in the submission form are also declared in your Financial Disclosure statement.	The authors have declared that no competing interests exist

Powered by Editorial Manager® and ProduXion Manager® from Aries Systems Corporation

View published research articles from *PLOS ONE* for specific examples.<br><br>**NO authors have competing interests**<br><br>Enter: *The authors have declared that no competing interests exist.*<br><br>**Authors with competing interests**<br><br>Enter competing interest details beginning with this statement:<br><br>*I have read the journal's policy and the authors of this manuscript have the following competing interests: [insert competing interests here]*<br><br>* typeset	
**Ethics Statement**<br><br>Enter an ethics statement for this submission. This statement is required if the study involved:<br><br>• Human participants<br>• Human specimens or tissue<br>• Vertebrate animals or cephalopods<br>• Vertebrate embryos or tissues<br>• Field research<br><br>Write "N/A" if the submission does not require an ethics statement.<br><br>General guidance is provided below. Consult the submission guidelines for detailed instructions. **Make sure that all information entered here is included in the Methods section of the manuscript.**	The retrospective study was approved by the Louisiana State University Health Sciences Center – New Orleans Institutional Review Board and received a waiver of informed consent for all patients studied. Patient data were deidentified and analyzed anonymously.

**Format for specific study types**<br><br>**Human Subject Research (involving human participants and/or tissue)**<br>• Give the name of the institutional review board or ethics committee that approved the study<br>• Include the approval number and/or a statement indicating approval of this research<br>• Indicate the form of consent obtained (written/oral) or the reason that consent was not obtained (e.g. the data were analyzed anonymously)<br><br>**Animal Research (involving vertebrate animals, embryos or tissues)**<br>• Provide the name of the Institutional Animal Care and Use Committee (IACUC) or other relevant ethics board that reviewed the study protocol, and indicate whether they approved this research or granted a formal waiver of ethical approval<br>• Include an approval number if one was obtained<br>• If the study involved *non-human primates*, add *additional details* about animal welfare and steps taken to ameliorate suffering<br>• If anesthesia, euthanasia, or any kind of animal sacrifice is part of the study, include briefly which substances and/or methods were applied<br><br>**Field Research**<br><br>Include the following details if this study involves the collection of plant, animal, or other materials from a natural setting:<br>• Field permit number<br>• Name of the institution or relevant body that granted permission	
**Data Availability**<br><br>Authors are required to make all data underlying the findings described fully available, without restriction, and from the time of publication. PLOS allows rare exceptions to address legal and ethical concerns. See the PLOS Data Policy and FAQ for detailed information.	Yes - all data are fully available without restriction

A Data Availability Statement describing where the data can be found is required at submission. Your answers to this question constitute the Data Availability Statement and **will be published in the article**, if accepted.<br><br>**Important:** Stating 'data available on request from the author' is not sufficient. If your data are only available upon request, select 'No' for the first question and explain your exceptional situation in the text box.<br><br>Do the authors confirm that all data underlying the findings described in their manuscript are fully available without restriction?	
**Describe where the data may be found in full sentences. If you are copying our sample text, replace any instances of XXX with the appropriate details.**<br><br>• If the data are **held or will be held in a public repository**, include URLs, accession numbers or DOIs. If this information will only be available after acceptance, indicate this by ticking the box below. For example: *All XXX files are available from the XXX database (accession number(s) XXX, XXX.).*<br>• If the data are all contained **within the manuscript and/or Supporting Information files**, enter the following: *All relevant data are within the manuscript and its Supporting Information files.*<br>• If neither of these applies but you are able to provide **details of access elsewhere**, with or without limitations, please do so. For example:<br><br>*Data cannot be shared publicly because of [XXX]. Data are available from the XXX Institutional Data Access / Ethics Committee (contact via XXX) for researchers who meet the criteria for access to confidential data.*<br><br>*The data underlying the results presented in the study are available from (include the name of the third party*	All relevant data are within the manuscript and its Supporting Information files.

and contact information or URL).<br>• This text is appropriate if the data are owned by a third party and authors do not have permission to share the data.<br><br>* typeset	
Additional data availability information:	Tick here if your circumstances are not covered by the questions above and you need the journal's help to make your data available.

Powered by Editorial Manager® and ProduXion Manager® from Aries Systems Corporation

Manuscript

Click here to access/download;Manuscript;Plos One Multi-Surge.docx

Multi-surge comparison of COVID-19 characteristics and outcomes of hospitalized patients in Louisiana

Covid-19 surge comparison and outcomes

Katie Taylor[1,2,¶]; Evan Rivere[1,2, ¶,*]; Tonya Jagneaux[1,2,&]; Gabrielle LeBoeuf[1,&], Karen Estela[1,&], Christi Pierce[1,&]; Catherine O'Neal[1,2¶];

Author Affiliations:

1. Franciscan Missionaries of Our Lady Health System, Baton Rouge, LA, USA
2. Louisiana State University Health Sciences Center, Baton Rouge, LA, USA

Word Count: 2958

* Corresponding Author: erive3@lsuhsc.edu (ER)

¶ These authors contributed equally to this work

& These authors also contributed equally to this work

Abstract:

Background

Peer reviewed data describing SARS-CoV-2 Omicron variant symptoms and clinical outcomes as compared to prior surges in the United States is thus far limited. We sought to determine disease severity, presenting features, and epidemiologic factors of the SARS-CoV-2 Omicron variant compared to prior surges.

Methods

Retrospective cohort analysis was performed on patients admitted during all five surges in Louisiana between March 2020 and January 2022. Patient data was pulled from the medical record, including a subset of patients during Surge 5 for individual chart review. Patients who were admitted to an FMOLHS hospital with a positive SARS-CoV-2 test during the 5 defined surge periods were included. Surges were compared using chi-squared tests and one way ANOVA for age, sex, vaccination status, length of stay, ICU status, ventilation requirement, and disposition at discharge. The records of patients admitted during the omicron surge were analyzed for presenting symptoms and incidental *SARS-CoV-2* diagnosis.

Results

With each subsequent surge, a smaller proportion of patients presenting to the ED were admitted. Patients admitted during surge 5 had shorter lengths of stay and fewer comorbidities than prior surges. Fewer patients in surge 5 presented with a respiratory condition and fewer required ICU admission. In surges 4 and 5, fewer vaccinated patients were admitted compared to their unvaccinated counterparts.

Overall mortality was lower in S5 (9%) than in S4 (15%). Of the SARS-Cov-2 positive patients in S5, 22.3% were felt to be incidental diagnoses.

Conclusions

As the COVID-19 pandemic progressed, a younger and less vaccinated population was at higher risk for severe disease, fewer patients required ICU admission and overall mortality decreased. Vaccinations have been protective for overall risk of hospitalization but once admitted did not confer additional protection against severe illness during the omicron surge. Age also played an important role in patient outcomes.

Introduction:

The COVID-19 pandemic has been characterized by surges of increased disease activity with intervening periods of reduced activity. The cause of this pattern is complex, as many factors and their interplay influence disease activity: societal and individual behavior, including variability in the use of mitigation; immune status of the population, either vaccinated, previously infected or both; and evolution of the virus, resulting in variants of varying transmissibility and virulence. As a result of these factors and their interactions, each surge has had a unique impact on healthcare systems and outcomes.

Recent surges, both globally and nationally, have been characterized by overwhelming spread of the Delta and, most recently, Omicron variants. The Omicron (*B.1.1.529*) variant was first detected in the United States on December 1, 2021 [1,2], and, by the week ending December 25, 2021, it became the predominant strain nationwide [3,4]. Cases due to Omicron have been reported to be less severe; however, infections with Omicron in previously recovered and/or fully vaccinated patients have been described, raising concerns of a larger susceptible population [5,6]. Data from South Africa comparing surges revealed a younger population with fewer admissions and a decreased need for respiratory care during the Omicron-predominant period [7]. In one US hospital's review of Omicron vs Delta surges, the former was associated with lower inpatient mortality; however, no difference was seen in outcome during the Omicron surge with regards to vaccination status [8]. We performed a retrospective cohort analysis of patients admitted to member hospitals of a Louisiana healthcare system during various surges of COVID-19. Our analysis compared the clinical

characteristics and outcomes between surges and the effect of vaccination on outcomes between the Delta and Omicron predominant surges. We also describe presenting symptoms of pediatric and adult patients admitted to our tertiary referral center during the Omicron predominant surge.

Materials and Method

The Franciscan Missionaries of Our Lady Health System (FMOLHS) includes five hospitals that serve as regional referral centers in Louisiana and Mississippi. Four hospitals that are located throughout Louisiana share a common medical record and were included in the analysis. COVID-19 admissions and emergency department (ED) visits during the time periods corresponding to the individual surges of COVID-19 activity within the state of Louisiana were reviewed. Five periods of increased activity were identified as surges: March 18, 2020 to May 1, 2020 (S1); July 3, 2020 to August 24, 2020 (S2); November 28, 2020 to January 30, 2021 (S3); July 10, 2021 to September 25, 2021 (S4, Delta); and the current surge December 15th 2021 to January 13, 2022 (S5, Omicron). These dates corresponded to the inflection point of increasing and decreasing patient visits to the hospital, except for the Omicron surge in which data analysis was stopped mid-January to compile results. Widespread sequencing was not performed during the first three surges, however based on public health data* it is assumed that the initial surges were a result of the ancestral COVID-19 variant. The Delta variant became the predominant strain in the United States during the summer of 2021. At the peak of this surge, 98.13% of all Louisiana test isolates were identified as the Delta variant.* In the winter

of 2021, Omicron became the dominant strain in Louisiana, accounting for 98.99% of sequenced strains at the surge's peak.*

This retrospective study was approved by the Louisiana State University Health Sciences Center – New Orleans Institutional Review Board and received a waiver of informed consent for all patients studied. We defined a COVID-19 diagnosis as any test positive for *SARS-CoV-2* documented within the electronic health record. Patients were only included if the positive test was performed during an ED visit or hospital admission within one of the surge periods. *SARS-CoV-2* testing was not required for all admissions but was recommended for any patient presenting with symptoms consistent with COVID-19, for patients undergoing an aerosol generating procedure and for patients admitted to mental health locations. Length of stay was calculated by the discharge date, date of death, or by the date when final data collection occurred if the patient was still admitted at that time. The remainder of the data points used for surge-to-surge comparison were obtained through reports generated through the institutions' shared electronic medical record.

Comparison of the surges was performed using excel and chi-squared tests for categorical variables and one-way analysis of variance (ANOVA) for numeric variables. Variables compared included age, sex, vaccination status, length of stay, ICU status, intubation/ventilation requirement and disposition at discharge. For analysis, we classified patients as unvaccinated, overdue for booster, or vaccinated. Unvaccinated patients had no record of vaccination or were partially vaccinated, having received 1 of 2 doses of a primary vaccine series. Patients overdue for booster received a 1 or 2 dose vaccine series and were eligible for an additional immunization but had not yet received the additional dose. Fully

vaccinated individuals completed all doses recommended at the time of admission or were not yet due for an additional dose.

A subset of patients admitted to FMOLHS's tertiary referral hospital between December 15th and January 7th, 2022, was abstracted for admitting symptoms. Symptoms were categorized as respiratory, gastrointestinal, neurologic, and/or cardiac. Admit diagnosis was noted. Patients could present with multiple symptoms if documented by the admitting physician. Sepsis was defined as suspected infection and 2 SIRS criteria with at least one criterion being either a qualifying white blood cell count or temperature. Septic shock was defined as sepsis with the need for vasopressor or fluid support to improve hypotension. Patients admitted for surgery, trauma, psychiatric illness, and/or patients whose test-based diagnosis *SARS-CoV-2* infection did not contribute to the reason for hospital admission were categorized as incidental infections.

Results

Surge-to-surge comparison

With each subsequent surge, there was a significant decrease in the proportion of COVID-19 patients presenting to the ED who were admitted to the hospital (51%, 43%, 38%, 20%, 15% for S1-S5 respectively, $p < .0005$) (Table 1). Though admission rates were highest in earlier surges, the later surges (S4 and S5) saw the largest number of *SARS-CoV-2* positive ED visits with S4 having the largest total number of hospital admissions.

Table 1. Comparison of characteristics and outcomes in all patients admitted with COVID-19 over the pandemic							
	No. (%)						
	Surge 1[a]	Surge 2	Surge 3	Surge 4	Surge 5	*p* value - all surges	*p* values - surge 4 vs 5
ED COVID-19 patients	1312	2462	3250	7570	5233		
**Admitted**	672 (51)	1058 (43)	1227 (38)	1522 (20)	787 (15)	<.0005	<.0005
Sex, M	340 (50)	530 (50)	617 (50)	788 (52)	395 (50)	.599	.120
Age, median (Q1, Q3)	67 (57,77)	66 (52,76)	69 (57,79)	58 (42,71)	62 (37,74)	<.0005	<.0005
Length of stay (days), median (Q1, Q3)	6 (3,12)	5 (2,10)	5 (3,11)	5 (2,10)	3 (2,17)	<.0005	<.0005
Patients with comorbidities[b]	665 (99)	1029 (97)	1179 (96)	1483 (97)	489 (62)	<.0005	<.0005
Respiratory condition on admit	381 (57)	681 (64)	742 (60)	948 (62)	332 (42)	<.0005	<.0005
ICU admissions	423 (62)	444 (42)	454 (37)	511 (34)	194 (25)	<.0005	<.0005
Age, ICU admits median (Q1, Q3)	67 (58,76)	65 (53,74)	67 (55,77)	57 (43,69)	63 (42,72)	<.0005	0.593
Ventilated	181 (27)	138 (13)	159 (13)	214 (14)	57 (7)	<.0005	<.0005
**Vaccination status**							
Unvaccinated				1287 (85)	546 (69)		<.0005
Fully vaccinated[c]				235 (15)	86 (11)		
Overdue for booster[d]				0	155 (20)		
**Disposition**							
Home	273 (40)	692 (65)	784 (64)	1100 (72)	588 (74)	<.0005	<.0005
Care facility	199 (30)	211 (20)	245 (20)	198 (13)	84 (11)		
Expired	200 (30)	155 (15)	198 (16)	224 (15)	69 (9)		
Not yet discharged					46 (6)		
Age of Expired median (Q1, Q3)	73 (62,82)	75 (66,84)	76 (69,82)	64 (54,75)	69 (61,80)	<.0005	0.026

[a]Surge 1: March 18,2020-May 1, 2020; Surge 2: July 3,2020-August 24, 2020; Surge 3: November 28, 2020-January 30, 2021; Surge 4: July 10, 2021-September 25, 2021; Surge 5: December 15, 2021- January 13, 2022

[b]Comorbidities include diabetes, heart conditions, hypertension, chronic kidney disease, chronic liver disease, chronic pulmonary conditions, and cancer

[c]Fully vaccinated is defined as up to date with primary COVID-19 vaccine series (and booster if recommended) at time of admission.

[d]Overdue for booster is defined as having received a full 1 or 2 dose primary COVID-19 vaccine series but overdue for additional dose(s) at the time of admission.

Patients admitted during S4 and S5 were significantly younger than those admitted during previous surges (median age [Q1, Q3]: 67[57, 77] (S1), 66[52, 76] (S2), 69[57, 79] (S3), 58[42, 71] (S4), and 62[37, 74] (S5), $p < .0005$) (Table 1). Hospital length of stay was significantly lower in S5 compared to previous surges (median [Q1, Q3]: 6[3, 12], 5[2, 10], 5[3, 11], 5[2, 10], 3[2, 7] days for S1-S5 respectively, $p < .0005$). The number of admitted patients having comorbidities decreased significantly during S5 compared to the previous surges (62% vs >96% S1-S4, $p < .0005$) and the proportion of patients presenting with a respiratory condition was also significantly lower in S5 vs previous surges (42% vs 57-64% in S1-S4, $p < .0005$). Significantly fewer patients required an ICU stay in S5 compared to previous surges (25% compared to 34% in S4 and 62% in S1, $p < .0005$). S4 was associated with the youngest median age at ICU admit and youngest age of death (ICU: 67, 65, 67, 57, 63 for S1-S5 respectively, $p < .0005$; mortality: 73, 75, 76, 64, 69 for S1-S5 respectively, $p < .0005$). The number of vaccinated individuals differed between S4 and S5 with a significantly higher number of patients admitted who were unvaccinated in S4 compared to S5 (85 vs 69%, $p < .0005$). Disposition type differed significantly between surges. S1 had the lowest percentage of patients discharged home (40%, $p < .0005$) and the largest percentage of patients who expired during their hospital stay (30%, $p < .0005$) (Table 1).

Adults in the Delta vs Omicron surge comparison by vaccination status

The clinical characteristics and outcomes amongst vaccinated and unvaccinated patients admitted during the Delta (S4) and the Omicron (S5) surges were compared (Table 2). Overall,

there were significantly fewer fully vaccinated patients admitted compared to unvaccinated during both surges (17% vs 83% (S4); 11% vs 66% (S5), p .0005). During S4, unvaccinated patients who were admitted to the hospital, admitted to the ICU, and those who died were significantly younger than vaccinated patients (57 vs 74 (p < .0005); 57 vs 74 (p<.0005); 62 vs 78 (p<.0005), respectively). This age difference was again seen in the S5 surge with the median age of fully vaccinated individuals and of overdue for booster individuals admitted to the hospital being higher than unvaccinated individuals (66 and 68 vs 63, p < .0005). The median age of vaccinated and overdue for booster patients was also higher than that of unvaccinated patients admitted to the ICU during the S5 surge (65, 66, 63 respectively, p .03). There was no difference in age by vaccination status for inpatient mortality during S5 (p. 0.29), although the overall mortality was lower in S5 (15% vs. 9 %, p <0.005). Length of stay was significantly different between vaccinated and unvaccinated individuals in S4 (4 vs 5 days, p .004) but did not reach clinical significance in S5 (4 days fully vaccinated, 4 days unvaccinated vs 5 days overdue for booster, p .06).

Table 2. Adults ≥ 18
Delta and Omicron surge data by vaccine status

	Surge 4 (Delta variant)<br>Total population<br>Total patients admitted, 1388<br>N (%)			Surge 5 (Omicron variant)<br>Total population<br>Total patients admitted, 688<br>N (%)			
Vaccine status	Vaccinated[a]<br>234 (17)	Unvaccinated<br>1154 (83)	P value<br><0.005	Vaccinated[a]<br>79 (11)	Unvaccinated<br>454 (66)	Overdue for Booster[b]<br>155 (23)	P value<br><.0005
Age, yrs<br>Median (Q1, Q3)	74<br>(66, 83)	57<br>(45, 69)	<.0005	66<br>(56, 74)	63<br>(45, 74)	68<br>(62, 78)	<.0005
Sex, M	123 (53)	584 (51)	.58	41 (51)	223 (49)	78 (50)	.95
Patients with comorbidities[c]	225 (96)	1131 (98)	.085	54 (68)	295 (65)	88 (57)	.119
Length of stay<br>Median (Q1, Q3)	4, (3, 9)	5 (3, 10)	.004	4<br>(2,5)	4<br>(2,8)	5<br>(2,9)	.06
**ICU status**	66 (28)	404 (35)	.04	16 (20)	108 (28)	43 (28)	.41
Age, ICU admits	74	57	<.0005	65	63	66	.03

Median (Q1, Q3)	(70, 86)	(44, 68)		(57,72)	(59,73)	(59, 77)	
Ventilated	17 (7)	185 (16)	<.0005	5 (6)	33 (7)	15 (10)	.88
**Disposition**							
Home	141 (60)	831 (72)	<.0005	64 (81)	327 (72)	105 (68)	.50
Care Facility	55 (24)	140 (12)		8 (10)	53 (12)	21 (14)	
Expired	38 (16)	183 (16)		4 (5)	46 (10)	19 (12)	
Not yet discharged				3 (3)	28 (6)	10 (6)	
Age of Expired Median (Q1, Q3)	78 (71, 86)	62 (52, 72)	<.0005	73 (70, 76)	67 (58, 80)	71 (66, 82)	.29

[a]Vaccinated is defined as up to date with primary COVID-19 vaccine series (and booster if recommended) at time of admission.

[b]Overdue for booster is defined as having received a full 1 or 2 dose primary COVID-19 vaccine series but overdue for additional dose(s) at the time of admission.

[c]Comorbidities include diabetes, heart conditions, hypertension, chronic kidney disease, chronic liver disease, chronic pulmonary conditions, and cancer

A higher percentage of patients required ventilation during S4 compared to S5 (14% vs 7%, p <.0005). Though vaccinated patients required less ventilatory support in S4 surge (7% vaccinated vs 16% unvaccinated, p< .0005), there was no difference in ventilatory support by vaccination status in S5. The percent of patients with comorbidities did not differ by vaccination status in either surge.

Omicron patient characteristics

In *SARS-CoV-2* positive patients during the Omicron surge (S5), respiratory complaints were present in 64% of adults, with gastrointestinal and neurologic symptoms occurring in greater than 20% of patients. 25.8% of adult patients presented with sepsis and 10.4% of adult patients presented with septic shock (Table 3).

Table 3. Characteristics of pediatric and adult patients admitted with Sars-CoV-2 during the Winter 2021-22 Surge		
N (%)		
Patients	Total population<br>≤17<br>65	Total population<br>≥18<br>337
Age, yrs (median)	1	63
Sex, M	34 (52)	170 (50)
Length of stay	2	3.1
ICU admissions	14 (21.5)	63 (18.6)
Fully Vaccinated	0	46 (14)
Ventilated	2 (3)	16 (5)
Mortality	0	9 (2.7)
**Presenting signs/symptoms**		
Respiratory	34 (52)	215 (64)
Gastrointestinal	23 (35)	76 (22.5)
Cardiac	0	42 (12.5)
Neurologic	4 (6)	75 (22.3)
Sepsis	24 (37)	87 (25.8)
Septic Shock	3 (5)	35 (10.4)
Coagulopathy	0	23 (7)
Incidental covid	11 (7)	79 (23.4)

Definition of signs/symptoms: respiratory – cough, stridor, shortness of breath, respiratory distress, infiltrates on chest film, hypoxia or hypoxemia; cardiac – chest pain, arrythmia, myocarditis, pericarditis, myocardial infarction, heart failure exacerbation; gastrointestinal – nausea, vomiting, diarrhea, appendicitis, gastrointestinal bleed; coagulopathy – arterial or venous thrombosis involving any organ system; sepsis – 2 SIRS criteria met with at least 1 criterion being either white blood cell count or temperature; septic shock – sepsis with hypotension requiring vasopressor or fluid support

In the S5 *SARS-CoV-2* positive pediatric population, 21.5% of children required an ICU admission with 3% of children requiring mechanical ventilation. The most common presenting symptom among pediatric patients was respiratory (52%) followed by gastrointestinal (35%). Only 7% of pediatric admits were found to be incidental diagnoses compared to 23% of adult admissions (Table 3).

Within the group of *SARS-CoV-2* positive patients, 5 (1%) patients were diagnosed with appendicitis of which 4 were between the ages of 11 and 16, and 11 (3%) patients presented with seizure, 2 of which were under the age of 1, and 8 of which were new in onset.

Additionally, we noted 19 (5%) presenting with atrial fibrillation with rapid ventricular rate, 10 (2%) presenting with sickle cell vaso-occlusive crisis and 7 (2%) with gastrointestinal bleeding.

Discussion

The Omicron surge began in the United States in December 2021. Following this variant's introduction hospitals and emergency departments became overwhelmed with patients as daily death rate and hospitalization rates climbed, similar to previous COVID-19 surges. Although the Omicron surge resulted in lowest admission rates of all surges (15%), this was counterbalanced by it yielding the second-highest number of *SARS-CoV-2* positive ED visits. In all, S5 resulted in more inpatient admissions than S1, despite a truncated analysis due to data collection.

The surge-to-surge comparison revealed an overall younger population and the less vaccinated population at risk for severe disease as the pandemic progressed. ICU admissions, percent of patients requiring ventilation, and percent of patients who expired while admitted all decreased with subsequent surges. Several factors likely contribute to this finding. Importantly, the age of admission, a powerful predictor of outcomes, generally declined with successive surges. Also, as clinicians gained experience in caring for COVID-19 patients and therapy choices improved, evolving medical care likely influenced outcomes. As the proportion of patients who were vaccinated or immune by prior infection increased – and as this acquired immunity waned over time – the host response played an important role in influencing outcomes. In addition, we were not able to capture the number of patients who were diagnosed and treated at home once home testing and improved ambulatory therapy was

available. All these factors likely affected inpatient outcomes. In short, the relationship between each of these factors and outcome is more difficult to elucidate.

Changes in hospital practice also influenced certain clinical outcomes. For example, due to resource limitation during S4, the hospital policy allowed for up to 30 LPM of high-flow oxygen delivered by heated, humidified nasal cannula to be cared for on the general inpatient wards, thus underestimating the number of critically ill patients. In effect, to a degree, this altered standard of care during S4 uncoupled the relationship between severity of illness and location of admission. This was not a common practice during previous or subsequent surges, and it is unclear if this practice may have influenced other clinical outcomes (mortality, length of stay, etc). The percent of patients admitted with primary respiratory symptoms decreased as the pandemic progressed, and this decrease was most pronounced in S5 compared to previous surges. This may have been due to an evolution in presentation of COVID-19 in the vaccinated population however the number of incidental COVID-19 diagnoses made during S5 (>20%) may also have played a role. There were significantly more patients admitted during S5 who had no comorbidities, potentially indicating that our incidental number of COVID-19 diagnoses also increased over previous. Unfortunately, our individual chart review for incidental numbers only included S5 and we cannot fully compare incidental diagnoses from previous surges.

In comparing the five surges within our health system, two overarching observations were noted: vaccination is protective against severe disease and age plays a significant role in outcome severity. Vaccination was clearly protective for overall risk of hospitalization in the Delta and Omicron surge; however, vaccination did not appear to be associated with the same degree of protection against severe illness once admitted (defined as ICU admission,

mechanical ventilation, disposition, or death) during S5 compared to S4. The overall outcomes of patients in S5 were improved versus S4 with fewer patients discharged to a care facility and lower mortality. Thirty-one percent of patients admitted during the Omicron surge had received a primary series or a series plus booster compared to 15% of patients during the Delta surge. The variant type may have played a part in outcome, but the surge-to-surge comparison also reveals that overall vaccination status is significantly improved during the Omicron surge and may also have played a role in overall outcomes.

Age continued to be a defining factor for outcome in S5 as it was in S4. The age of those admitted with COVID-19 decreased as the pandemic progressed and may have been protective for some outcomes. There was a protective effect of younger age in disposition between vaccination groups in S4. More unvaccinated patients were able to be discharged home during S4; however, their median age was 17 years younger than their vaccinated counterparts. Despite the large age gap, mortality was similar between the younger unvaccinated patients and the more elderly, vaccinated population during Delta supporting the protective effect of the vaccine. A younger age was protective in all outcomes except mortality during S5. During the Omicron surge, vaccination appeared to trend towards more protection than age as the vaccinated group was only 3 years older than the unvaccinated group, and vaccinated patients were more likely to be discharged to home. However, this difference did not reach statistical significance.

Previous pediatric data collected from five pediatric hospitals during the Delta surge showed 29.5% of pediatric patients with COVID-19 required an ICU admission.(9) In the same study, the authors reported a 19% incidental diagnosis rate. In our review of pediatric Omicron

data, we also saw a >20% rate of ICU admissions in children with respiratory symptoms, gastrointestinal symptoms, and sepsis being the most common admission diagnoses. However, only 7% of pediatric cases were incidental suggesting that our admitted pediatric patient population during Omicron was more likely admitted with symptomatic illness.

Patients with acute symptomatic COVID-19 present most commonly with respiratory symptoms, but often multiple organ systems are involved in the disease and patients can occasionally present solely with non-respiratory symptoms [10–12]. In our adult population, gastrointestinal symptoms and sepsis were common presenting findings during S5. Previous literature supports links between COVID-19 and appendicitis, seizures, gastrointestinal bleeding, atrial fibrillation, and sickle cell vaso-occlusive crisis [13–21], and these associations were supported by our findings.

This study has several limitations. We did not have individual variant analysis for each patient and therefore cannot determine if patient outcomes are directly related to variant affect. We did not perform a multivariate analysis which may help us to determine the weight of individual factors associated with outcomes. A multivariate analysis would be difficult in this size study as age, vaccination and comorbidities may trend together in population subsets. Omicron data was collected near the peak of the surge and not through the end of the surge. Total admitted patients, length of stay and mortality are likely underrepresented when compared to surge 1 through surge 4, where data collection included the entire time interval of each surge. Thus, the numbers may be underpowered to find a significant difference.

Despite the limitations, this study reveals the challenges hospitals faced in anticipating patient care as each surge occurred. The differences between prognosis of patients in the Delta

vs Omicron surge were significant. However, with 25% of the Omicron admissions requiring ICU care, an older age of admission compared to Delta and 9% inpatient mortality at the time of our analysis, the severity of illness and anticipated effect of the Omicron variant on hospital capacity was initially underestimated. Our study reveals not only the immense resources that each surge has required but also the variety of patient presentations and diverse organ system specific care required for COVID-19 patients.

Conclusion

Subsequent surges with potentially new variants are an expected reality, leaving us with two observations: during increased community activity of *SARS-CoV-2*, acute care evaluation should include COVID-19 testing, given the variability of organ system involvement at presentation. In addition, despite differences in variant characteristics, hospitals should brace for high admission rates and mortality with subsequent surges.

Acknowledgments:

The authors thank Dr. Hollis O’Neal.

*Data obtained from correspondence with the Louisiana Department of Health

Bibliography

1. SARS-CoV-2 B.1.1.529 (Omicron) Variant — United States, December 1–8, 2021. MMWR Morb Mortal Wkly Rep 2021;70:1731-1734. DOI: http://dx.doi.org/10.15585/mmwr.mm7050e1external icon [Internet]. 2021 Dec. Available from: https://www.ncbi.nlm.nih.gov/sars-cov-2

2. Centers for Disease Control and Prevention. First Confirmed Case of Omicron Variant Detected in the United States [Internet]. 2021 Dec [cited 2022 Feb 10]. Available from: https://www.cdc.gov/media/releases/2021/s1201-omicron-variant.html

3. Classification of Omicron (B.1.1.529): SARS-CoV-2 Variant of Concern [Internet]. World Health Organization. 2021 [cited 2022 Feb 10]. Available from: https://www.who.int/news/item/26-11-2021-classification-of-omicron-(b.1.1.529)-sars-cov-2-variant-of-concern

4. Institute for Health Metrics and Evaluation. COVID-19 Projections [Internet]. Covid Data Tracker. 2022 [cited 2022 Feb 16]. Available from: https://covid19.healthdata.org/global?view=cumulative-deaths&tab=trend

5. Klaassen F, Chitwood MH, Cohen T, Pitzer VE, Russi M, Swartwood NA, et al. Population immunity to pre-Omicron and Omicron SARS-CoV-2 variants in US states and counties through December 1, 2021. 2022; Available from: https://doi.org/10.1101/2021.12.23.21268272

6. Accorsi EK, Britton A, Fleming-Dutra KE, Smith ZR, Shang N, Derado G, et al. Association Between 3 Doses of mRNA COVID-19 Vaccine and Symptomatic Infection Caused by the SARS-CoV-2 Omicron and Delta Variants. JAMA. 2022 Feb 15;

7. Maslo C, Friedland R, Toubkin M, Laubscher A, Akaloo T, Kama B. Characteristics and Outcomes of Hospitalized Patients in South Africa During the COVID-19 Omicron Wave Compared With Previous Waves. JAMA [Internet]. 2022 Feb 8;327(6):583–4. Available from: https://doi.org/10.1001/jama.2021.24868

8. Modes ME, Directo MP, Melgar M, Johnson LR, Yang H, Chaudhary ; Priya, et al. 2 (Delta) and B.1.1.529 (Omicron) Variant Predominance-One Hospital [Internet]. Available from: https://covid19.ca.gov/variants/

9. Wanga V, Gerdes ME, Shi DS, Choudhary R, Dulski TM, Hsu S, et al. Morbidity and Mortality Weekly Report Characteristics and Clinical Outcomes of Children and Adolescents Aged <18 Years Hospitalized with COVID-19-Six Hospitals, United States, July-August 2021 [Internet]. Available from: https://www.cdc.gov/coronavirus/2019-ncov/variants/delta-variant.html;

10. Pan L, Mu M, Yang P, Sun Y, Wang R, Yan J, et al. Clinical Characteristics of COVID-19 Patients With Digestive Symptoms in Hubei, China: A Descriptive, Cross-Sectional, Multicenter Study. Official journal of the American College of Gastroenterology | ACG [Internet]. 2020;115(5). Available from:

https://journals.lww.com/ajg/Fulltext/2020/05000/Clinical_Characteristics_of_COVID_19_Patients_With.25.aspx

11. Zhang C, Shi L, Wang FS. Liver injury in COVID-19: management and challenges. Lancet Gastroenterol Hepatol [Internet]. 2020/03/04. 2020 May;5(5):428–30. Available from: https://pubmed.ncbi.nlm.nih.gov/32145190

12. Shi S, Qin M, Shen B, Cai Y, Liu T, Yang F, et al. Association of Cardiac Injury With Mortality in Hospitalized Patients With COVID-19 in Wuhan, China. JAMA Cardiology [Internet]. 2020 Jul 1;5(7):802–10. Available from: https://doi.org/10.1001/jamacardio.2020.0950

13. Marasco G, Maida M, Morreale GC, Licata M, Renzulli M, Cremon C, et al. Gastrointestinal Bleeding in COVID-19 Patients: A Systematic Review with Meta-Analysis. Nakamura M, editor. Canadian Journal of Gastroenterology and Hepatology [Internet]. 2021;2021:2534975. Available from: https://doi.org/10.1155/2021/2534975

14. Elshazli RM, Kline A, Elgaml A, Aboutaleb MH, Salim MM, Omar M, et al. Gastroenterology manifestations and COVID-19 outcomes: A meta-analysis of 25,252 cohorts among the first and second waves. J Med Virol [Internet]. 2021/02/23. 2021 May;93(5):2740–68. Available from: https://pubmed.ncbi.nlm.nih.gov/33527440

15. Meyer JS, Robinson G, Moonah S, Levin D, McGahren E, Herring K, et al. Acute appendicitis in four children with SARS-CoV-2 infection. Journal of Pediatric Surgery Case Reports [Internet]. 2021;64:101734. Available from: https://www.sciencedirect.com/science/article/pii/S2213576620303687

16. Panepinto J, Brandow A, Mucalo L, Yusuf F, Singh A, Taylor B, et al. Coronavirus Disease among Persons with Sickle Cell Disease, United States, March 20–May 21, 2020. Emerging Infectious Disease journal [Internet]. 2020;26(10):2473. Available from: https://wwwnc.cdc.gov/eid/article/26/10/20-2792_article

17. Singh A, Brandow AM, Panepinto JA. COVID-19 in individuals with sickle cell disease/trait compared with other Black individuals. Blood Advances. 2021 Apr 1;5(7):1915–21.

18. Narula N, Joseph R, Katyal N, Daouk A, Acharya S, Avula A, et al. Seizure and COVID-19: Association and review of potential mechanism. Neurology, Psychiatry and Brain Research [Internet]. 2020;38:49–53. Available from: https://www.sciencedirect.com/science/article/pii/S0941950020301408

19. Keshavarzi A, Janbabaei G, Kheyrati L, Ghavamabad LH, Asadi-Pooya AA. Seizure is a rare presenting manifestation of COVID-19. Seizure [Internet]. 2021/01/23. 2021 Mar;86:16–8. Available from: https://pubmed.ncbi.nlm.nih.gov/33515905

20. Gawałko M, Kapłon-Cieślicka A, Hohl M, Dobrev D, Linz D. COVID-19 associated atrial fibrillation: Incidence, putative mechanisms and potential clinical implications. IJC Heart & Vasculature [Internet]. 2020;30:100631. Available from: https://www.sciencedirect.com/science/article/pii/S2352906720303298

21. Romiti GF, Corica B, Lip GYH, Proietti M. Prevalence and impact of atrial fibrillation in hospitalized patients with covid-19: A systematic review and meta-analysis. Vol. 10, Journal of Clinical Medicine. MDPI; 2021.
