# Supplementary material for: Clinical characteristics and outcomes of SARS-Cov-2 B.1.1.529 infections in hospitalized patients and multi-surge comparison in Louisiana
Source: PLoS One. 2022 Oct 21;17(10):e0268853. doi: 10.1371/journal.pone.0268853 (PMC9586372; doi:10.1371/journal.pone.0268853)
Supplement: Attachment — Submitted filename: Response to Reviewers 9-14-22.docx [file pone.0268853.s004.docx]

Author Response to Reviewers

*Please note that the lines referenced in the author comments refer to that of the revised manuscript with tracked changes and no longer correlate with the lines referenced by the reviewer comments as line numbers changed due to edits.*

Responses to Reviewer #2 Comment:

Reviewer #2: The comments have been addressed, however, I encouragely suggest to be more conditional in the conclusions in the abstract, since there are associations, but not way to prove causality, considering issues with the changes in vaccination rates and determination of virus variants. In the same way, to specify that all the cases admitted to the hospital were considered, to clarify that there is not a sample size calculation (and the implications of that to make statistical inference).

Author Response:

1. Abstract conclusion has been edited to make conclusions more conditional/associations and less causal.
   a. Revision (Lines 44-48): As the COVID-19 pandemic progressed, a younger and less vaccinated population was associated with higher risk for severe disease, fewer patients required ICU admission and overall mortality decreased. Vaccinations seemed to be protective for overall risk of hospitalization but once admitted did not seem to confer protection against severe illness during the omicron surge. Age also contributed to patient outcomes.
2. Methods section has been edited to clarify that sample size calculation was not performed.
   a. Revision (Lines 94-96): All COVID-19 admissions and emergency department (ED) visits during the time periods corresponding to the individual surges of COVID-19 activity within the state of Louisiana were reviewed and considered; no sample size calculation was performed.
